# Poly-Methyl Methacrylate/Polyvinyl Alcohol Copolymer Agents Applied on Diabetic Wound Dressing

**DOI:** 10.1038/s41598-017-10193-5

**Published:** 2017-08-25

**Authors:** Hsiao-Ting Hsieh, Hung-Ming Chang, Wei-Jhih Lin, Yao-Tsung Hsu, Fu-Der Mai

**Affiliations:** 1 Graduate Institute of Medical Science, College of Medicine, Taipei Medical University, No. 250 Wuxing St., Taipei, 11031 Taiwan; 2 Department of Biochemistry and Molecular Cell Biology, School of Medicine, Taipei Medical University, No. 250 Wuxing St., Taipei, 11031 Taiwan; 3 Department of Anatomy and Cell Biology, School of Medicine, Taipei Medical University, No. 250 Wuxing St., Taipei, 11031 Taiwan; 4 Department of Forensic Science, Central Police University, No.56, Shuren Rd., Guishan Dist., Taoyuan City, 33304 Taiwan

## Abstract

Due to the difficulty of healing chronic wound, in the process of changing dressing, secondary damage on the tissue caused by adhesion should be prevented. In this study, the new dressing of particle hydrogels synthesized with poly-methyl methacrylate and poly-vinyl alcohol precursors were proposed. In addition, cell safety tests, animal’s allergic stimulation, and animal’s wound healing experiments were conducted for particle hydrogels. On one hand, in L929 cell experiment, the results of particle hydrogels extract 3-(4,5-Dimethylthiazol-2-yl)-2,5-diphenyl tetrazolium bromide tests and lactate dehydrogenase test trial show that there are no safety concerns over particle hydrogels. On the other hand, New Zealand white rabbits were chosen for skin sensitization tests in animal trials, which show the consistent results. At last, wound healing tests used diabetes induction with 10-week-old rats and three-month-old Landrace pigs, with the tissue histology. In short, through this experiment, it is found that in the early phase of the diabetic rats and pigs’ wound healing, using particle hydrogels can enhance collagen formation, and achieve the goal of faster wound healing.

## Introduction

The world’s population is aging, and occurrence rates of obesity and chronic diseases are increasing. As the result, each system in the human body is impacted, leading to various chronic diseases and complications, among which ill care of chronic wounds can severely affect people’s quality of life. There are three types of chronic wounds: diabetic foot ulcers (DFUs), ulcers of the lower limbs, and bedsores. It usually takes more than 12 weeks for chronic wounds to heal^1, 2^. In addition, since chronic wound tissues cannot completely function or form tissues, wound care is mainly accomplished through infection control, wound cleaning, and maintaining a humid environment for wound healing^3, 4^.

Diabetes mmellitus (DM) is one of the primary diseases in Taiwan, among which type-II DM patients comprise 90%^5^. According to the World Health Organization’s definition of DFUs, the calf of a diabetic patient has ulcers and/or complicated nerve-related lesions, along with peripheral vascular diseases and damage or infection of deep tissues^6^. In addition, diabetic patients’ lesions of the lower limbs are caused by hyperglycemia. As the foot is an organ far from the heart, the occlusion is the most severe part, which gradually leads to foot edema, rotting, necrosis, or gangrene. Actually, DFUs are one of the primary complications, and one of the causes of amputation or patient death. According to statistical data, DFU patients comprise around 20% of diabetic patients, among which the rate of amputation caused by lower limb ulcers and gangrene is up to 40%^7, 8^.

In 1937, poly-methyl methacrylate (PMMA) was introduced to Taiwan to make dentures and be used as bone cement filling material^9^. PMMA is characterized by its compliance to the shape of the surrounding environment, and its implantation can provide a strong physical structure to fill in gaps. In this study, polyvinyl alcohol (PVA), which is applied as an adhesive, stabilizer, sensitizer, and filling material, was also used. Other studies mentioned that a mixture of PVA and chitin can through electro-spinning make wound dressings. Such wound dressing provides good biological compatibility, biodegradability, cell-binding ability, antimicrobial activity, and enhancement of wound healing^10, 11^. In this paper, PMMA and PVA were combined using an ultraviolet (UV) light crosslinking method to develop particle-based wound dressings.

## Results

### Particle hydrogels process

**Figure 1:**
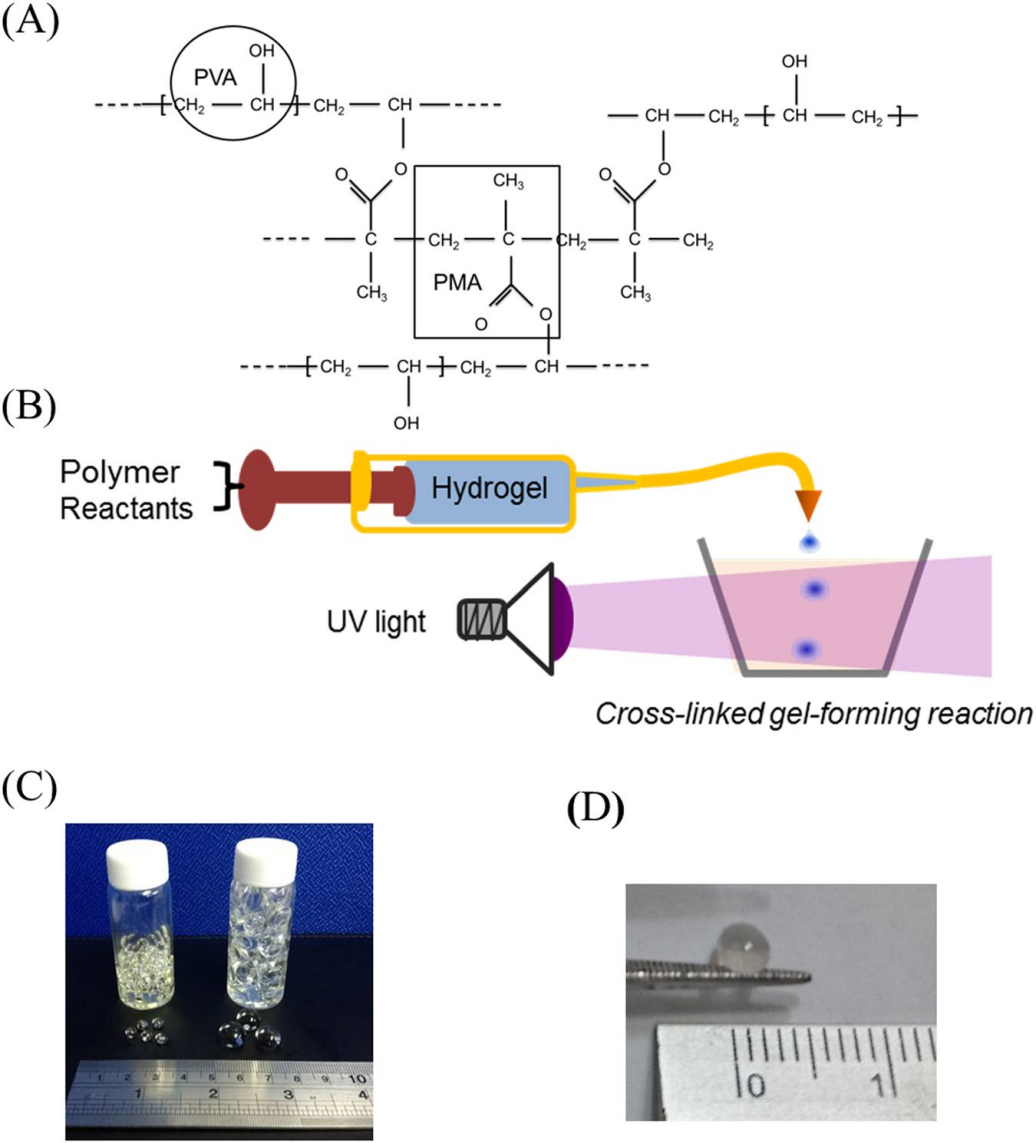
(**A**) Chemical structure of the particle hydrogel. (**B**) The process of particle hydrogels using UV light cross-linked gels to form a reaction. (**C**) and (**D**) Picture of the particle hydrogels.

In this study, PMMA and PVA were adopted as the precursors to form particle hydrogels (PHs) (Fig. 1A) through a crosslinking reaction with UV light (Fig. 1B). Actually, PHs has a reticular structure. In this process, the size of PHs can be regulated and controlled through regulating and controlling the diameter of the syringe. PHs was formed by this method were viscous and biocompatible, and the expansion degree and humidity were regulated and controlled.

In order to understand particle sizes of the PHs used in this study (Fig. 1C and D, Table 1), two PHs of different sizes were adopted to analyze the weight, number, and volume. Through testing, after in this study the PHs had absorbed moisture, a 30-fold expansion ratio in the volume and a 20-fold increase in the weight were found.

**Table 1:** Test of particle hydrogel weight and volume with 10- and 20-uL particles.

Precursor particle volume	Hydrogel weight (g/mL)	Volume (density 1.2)	Weight (g)	Moisture absorption rate (weight)	Number	Before size (mL)	After size (mL)	Moisture absorption rate (size)
10 uL	2.29	1.91	52.64	23.03	180	0.01	0.35	33.07
10 uL	2.16	1.80	50.30	23.32	165	0.01	0.31	28.37
10 uL	2.09	1.75	59.32	28.32	165	0.01	0.33	31.51
20 uL	2.05	1.71	48.22	23.56	55	0.03	1.00	32.24
20 uL	2.01	1.67	49.08	24.43	56	0.03	0.80	26.88
20 uL	1.97	1.64	48.04	24.37	61	0.03	0.74	27.40

### Cell toxicity of the L929 cell line co-cultured with particle hydrogel extracts

**Figure 2:**
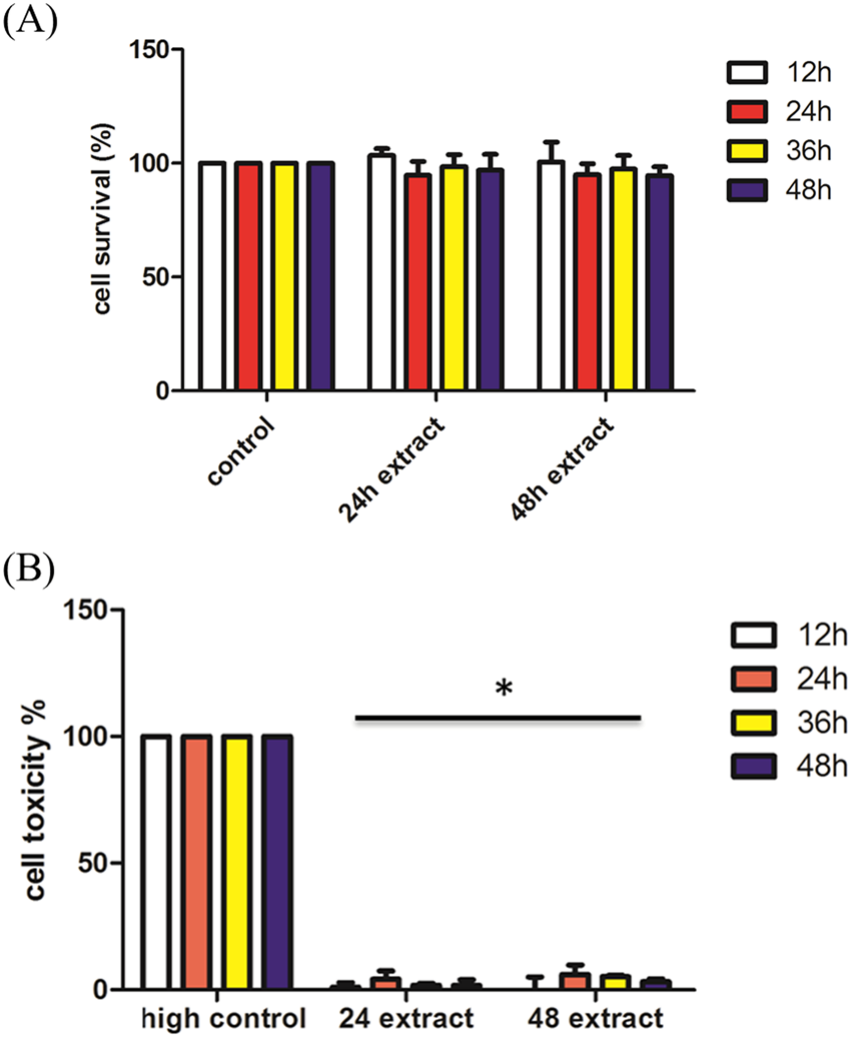
Cell toxicity of the L929 cell line co-cultured with particle hydrogel extracts. (**A**) MTT assay of L929 cells incubated for 12, 24, 36, and 48 hr with particle hydrogel extracts. (**B**) Lactate dehydrogenase (LDH) assay of L929 cells incubated for 12, 24, 36, and 48 hr with particle hydrogel extracts. *p < 0.05 compared with high control (100% death).

To evaluate the toxicity of the PHs, mouse fibroblast L929 cells and PH extracts were cultured together for 12, 24, 36, and 48hr. As MTT assay data (Fig. 2A) demonstrated, after cell strain L929 and PH extracts were cultured for 12, 24, 36, and 48hr, the L929 cell viability did not significantly decline, and this shows that the PH extracts did not affect cell viability.

Since L929 cell growth was not affected by the PH extracts, the PH extracts’ influence on cell toxicity was further surveyed. Through co-culturing of L929 cells with PH extracts, the content of LDH released by cells was determined. From the results of the cell toxicity trial (Fig. 2B), the amount of LDH released by the control group (with no PH extract) was the same as that of the experimental groups. The extremely low percentage of LDH release indicated that L929 cell membranes were not damaged by the PH extracts.

### Stimulation reaction of rabbit skin allergic of particle hydrogels

**Figure 3:**
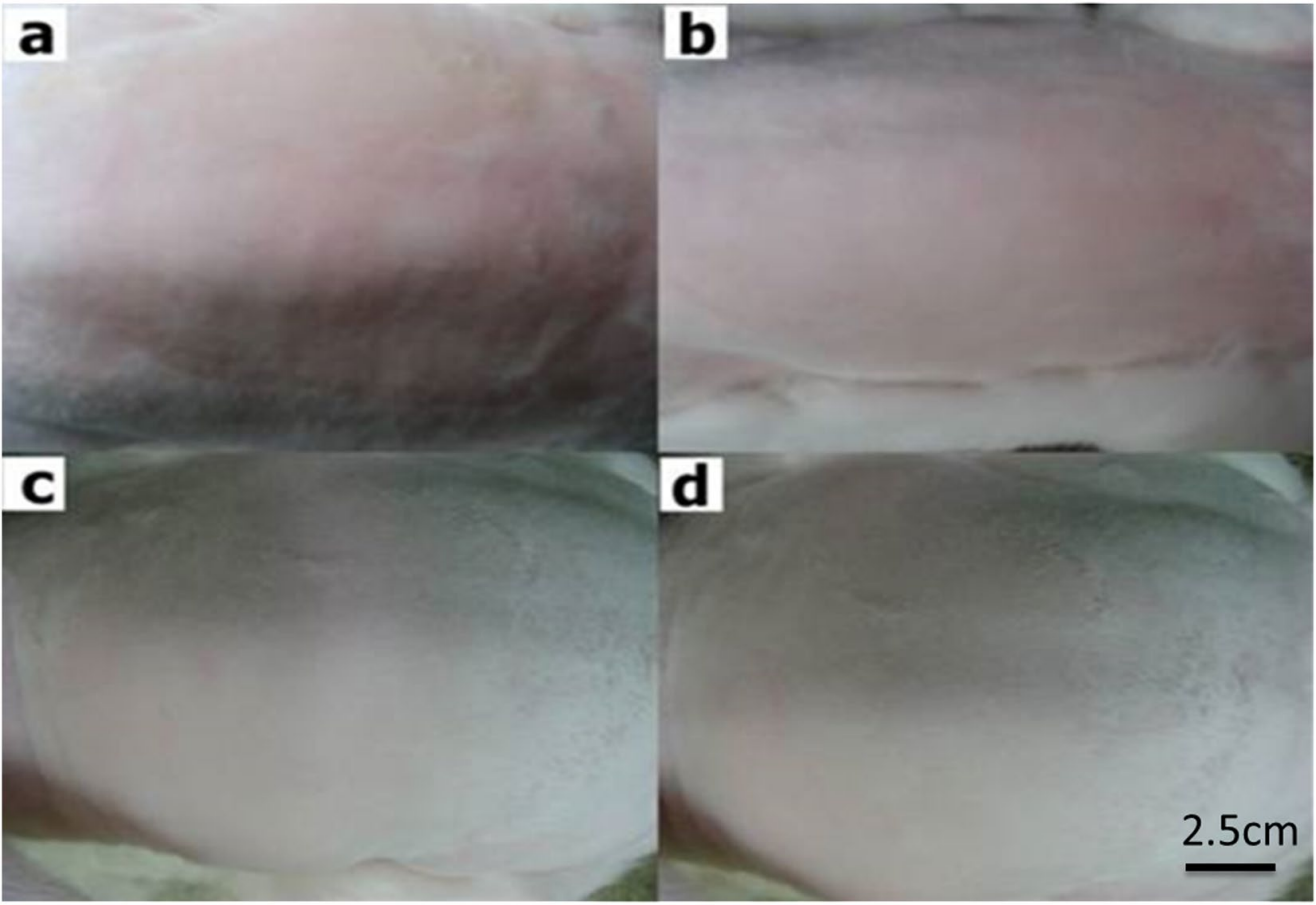
The back of a New Zealand White rabbit onto which three types of wound dressing were placed and observed (**a**) 0 hr, (**b**) 24 hr, (**c**) 48 hr, and (**d**) 72 hr. The scale bar is 2.5 cm.

In order to confirm the allergic stimulation reaction caused by PHs used on the skin, white rabbits with fur removed and skin disease on the back were observed for 24, 48, and 72hr (Fig. 3A–D), while the commercial product, Intrasite was used as a comparison item, with observations of skin redness and swelling. As shown in Table 2, the PHs caused no skin allergy or skin stimulation.

**Table 2:** Scores of erythema, eschar, and edema formation.

Check point/Sample		Erythema Value	Oedema Value
24 hr	Control	0	0
	Gauze	0	0
	Intrasite gel	0	0
	Hydrogel	0	0
48 hr	Control	0	0
	Gauze	0	0
	Intrasite gel	0	0
	Hydrogel	0	0
72 hr	Control	0	0
	Gauze	0	0
	Intrasite gel	0	0
	Hydrogel	0	0

### Cell toxicity of L929 cells co-cultured with particle hydrogel extracts of collagen, silver nanowire, and chitosan

**Figure 4:**
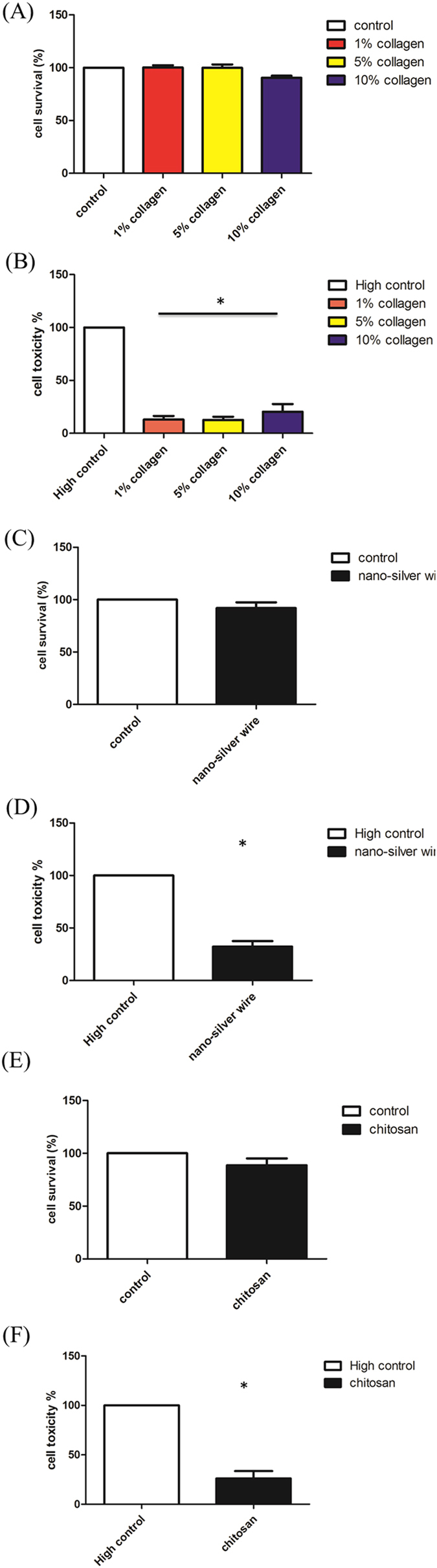
Cell toxicity of L929 cells co-cultured with particle hydrogel extracts of collagen, silver nanowire, and chitosan. L929 cells were incubated for 24hr with 1%, 5%, and 10% collagen particle hydrogel extracts and then examined by an MTT assay (**A**) and lactate dehydrogenase (LDH) assay (**B**). L929 cells were incubated for 24 hr with silver nanowire particle hydrogel extracts and then examined by an MTT assay (**C**) and LDH assay (**D**). L929 cells were incubated for 24 hr with 5% chitosan particle hydrogel extracts and then examined by an MTT assay (**E**) and LDH assay (**F**). *p < 0.05 compared with high control.

In order to achieve the goal of speeding up wound healing, collagen, silver nanowire, and chitosan were chosen as additives of the PH compounds. We adopted the same extract assay method to conduct MTT (Figs 4A,C,E) and LDH (Figs 4B,D,F) trials, and results showed that adding collagen, silver nanowire, and chitosan to the PH compounds did not give rise to cell toxicity reactions.

### Wound healing of the STZ-induced diabetes Wistar rats

**Figure 5:**
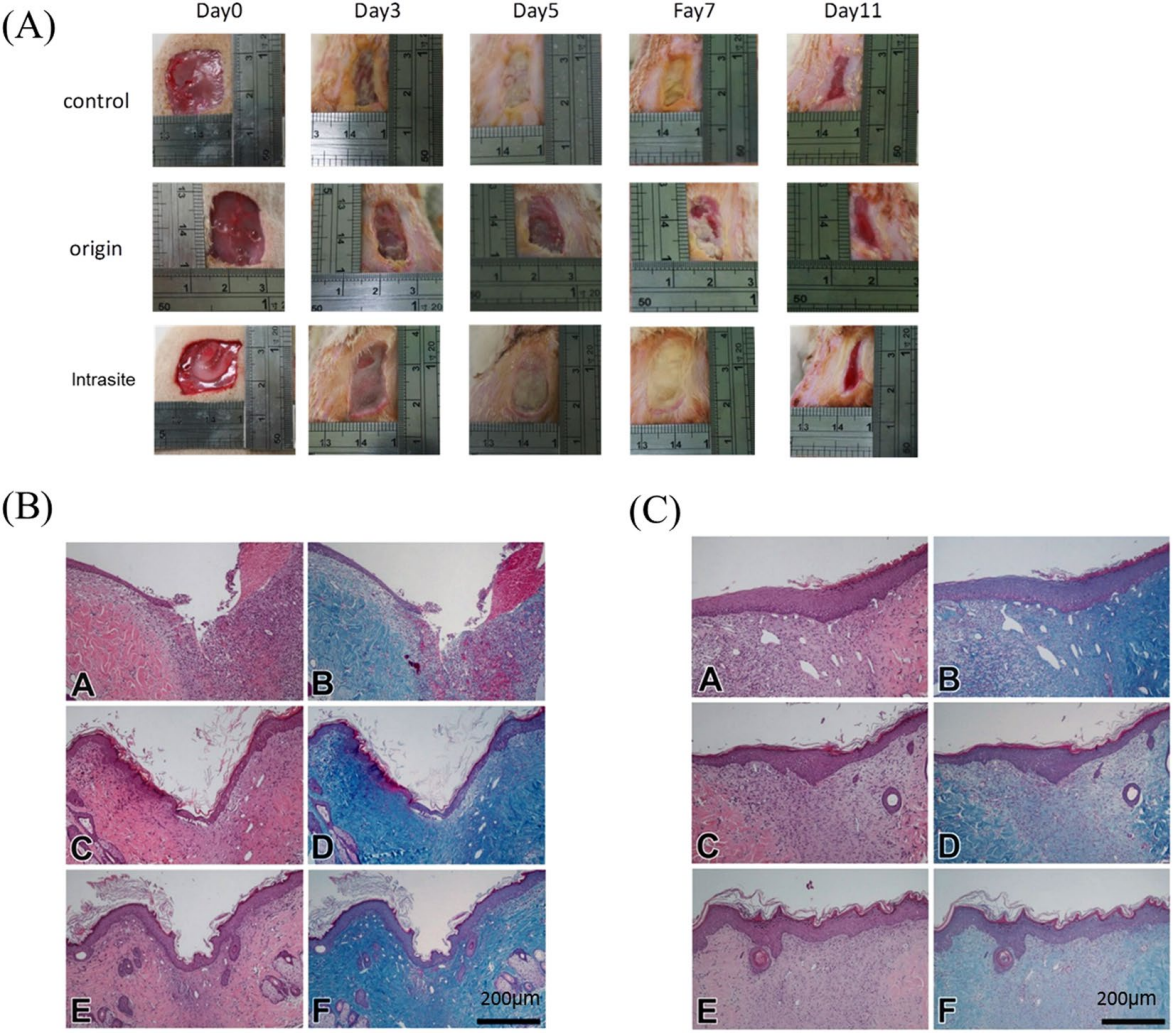
Diabetes mellitus was induced in Wistar rats, and then 2 × 2-cm wounds were created to observe wound healing. (**A**) The wound area size of the control group, original particle hydrogels group, and Intrasite group (a commercial product) at day 0, day 3, day 5, day 7 and day 11. (**B**) A 7th-day tissue section stained with H&E (control for A, original particle hydrogels for C and Intrasite for E) and Masson’s trichrome (control for B, original particle hydrogels for D and Intrasite for F). The scale bar is 200 micrometer. (**C**) A 14th-day tissue section stained with H&E (control for A, original particle hydrogels for C and Intrasite for E) and Masson’s trichrome (control for B, original particle hydrogels for D and Intrasite for F). The scale bar is 200 micrometer.

In the animal wound model, wound healing of Wistar rats with STZ-induced diabetes and wounds on both sides of their backs was observed for a period of 14 days. On the 7th day, wound-healing conditions of the diabetic rats (Fig. 5A) without using PHs of the same as those of diabetic rats with PHs. The wound-healing conditions did not significantly differ from those of rats using the commercial product such as the wound scabs cover the wound. Such wound-healing conditions remained the same to the end of the observation period. Rats were sacrificed on days 7 (Fig. 5B) and 14 (Fig. 5C), and sliced tissues were analyzed by HE and Masson’s trichrome staining. On day 7, sliced diagrams showed that after using PHs, in the aquamarine blue area represented collagen structure by Masson’s trichrome, the collagen accumulation around the rats’ wounds was better than that without using PHs and that using the commercial product. Then, on day 14, collagen accumulation in the PH group was less, yet the distribution was more even on connective tissues in the dermis, reflecting the fact that PHs can trigger production of collagen in the early stage.

### Wound healing of the STZ-induced diabetic pigs

**Figure 6:**
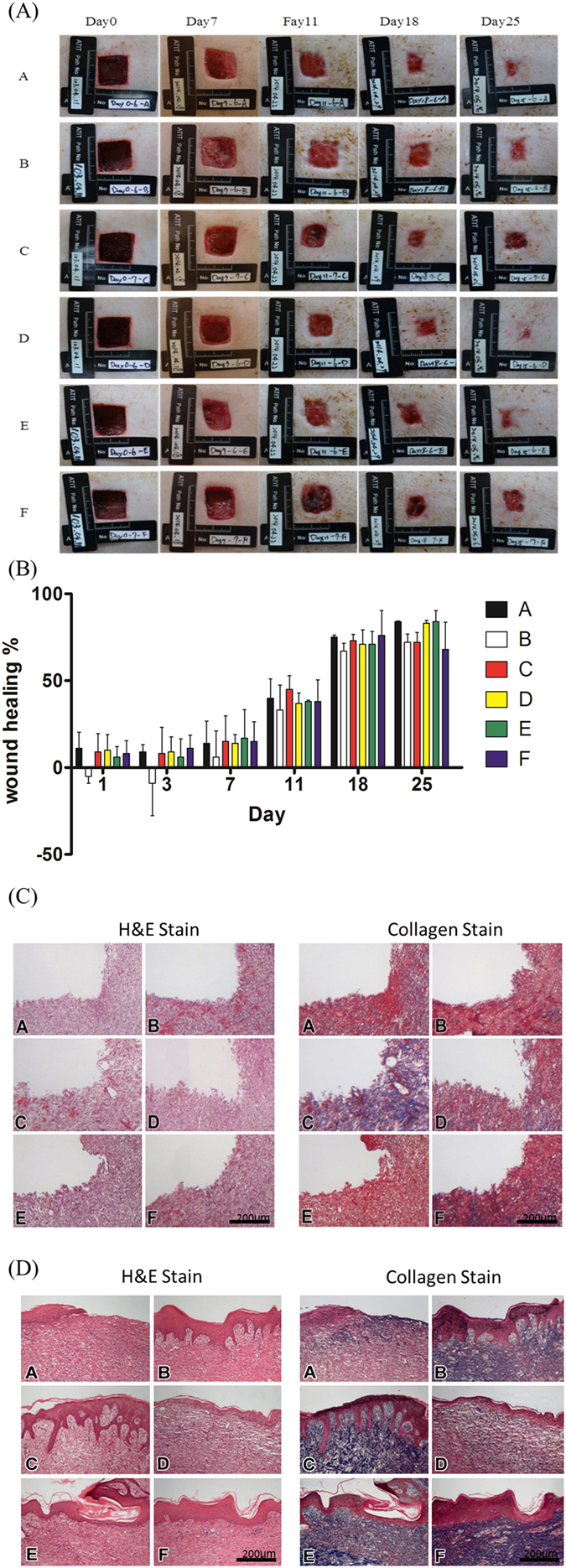
Diabetes mellitus was induced in Landrace pigs, and 2 × 2-cm wounds were created to observe wound healing. (**A**) The wound area size of the original type, original type mixed with 5% collagen, original type mixed with 350-ppm silver nanowire, original type mixed with 175-ppm silver nanowire, original type mixed with 5% collagen, 5% chitosan, and 350-ppm silver nanowire, and original type mixed with 5% collagen, 5% chitosan, and 175-ppm silver nanowire at day 0, day 7, day 11, day 18 and day 25. (**B**) The wound healing area quantitative analysis. (**C**) A 10th-day tissue section stained with H&E stain and Masson’s trichrome stain. The scale bar is 200 micrometer (**D**) A 21st-day tissue section stained with H&E stain and Masson’s trichrome stain. The scale bar is 200 micrometer.

This study focused on discussing diabetic pigs’ wound healing since human’s skin thickness and composition are close to those of pigs, and it was found in the previous rat experiment that PHs can accelerate wound healing in the early stage of diabetic disease. Wound-healing conditions were measured on days 0, 7, 11, 18 and 25 (Fig. 6A). In the first 7 days, pigs’ wound healing in each group did not significantly differ. However, after day 11, the wound healing areas were significantly reduced its area (Fig. 6B). Figure 6C and D present a tissue slice analysis of H&E staining of nuclei and Masson’s trichrome staining of collagen. On day 10 (Fig. 6C), it was observed that compared to group A, more collagen had aggregated on the wound, signifying that such wound dressings can induce collagen expression in the wound inflammation stage. By day 21 (Fig. 6D), collagen expression had taken place in each group, implying that after being covered by wound dressings for a long period of time, the PHs had induced collagen expression in the wound.

## Discussion

Wound dressings can be classified into various types, such as cheap gauze dressings that can be easily applied to all types of wounds. Transparent cover film can be utilized on the wound for one week to prevent abrasion of the wound, keep the wound dry, avoid bacterial contamination, and permit easy observation^12, 13^. Gel-based dressings have very strong adhesion and high absorbability^14^. Our dressings allow observation of the wound as easily as transparent films and have high absorbability as do gel-based dressings. Meanwhile, they can be applied to larger-area or deeper wounds by controlling the size of PHs, and their high absorbability allows them adhere to the wound without falling off before the particle absorb excess tissue fluid. Moreover, since PHs is not viscous when expanding, it is easy to flush and remove PHs with saline solution, which effectively decreases the secondary damage resulting from dressing replacement during wound healing^15^.

Diabetic ulcers are a common complication among diabetic patients. Due to slow wound healing, in order to control deterioration of DFUs, multidimensional treatment must be used, including oral antibiotics, prevention or restriction of infective progress and use of dressings that can stimulate wound healing. A good wound dressing is critical for diabetic ulcers, because it can absorb any surplus water content in the wound, while triggering dermal cell migration and regeneration. Moreover, a good dressing can promote expression of collagen, raise biocompatibility, and decrease the frequency of dressing changes^16, 17^.

In this study we use Wistar rats and Landrace pigs to be our animal skin wound models. In Wistar rats experiment progress we found in early stage (5^th^ and 7^th^ day) the skin wound covered the scabs that cause the skin wound look like healing but in the tissue sections the original particle hydrogels and Intrasite group had more complete epidermis and more collagen expression.

In this study, PHs is with different contents used. Among them, one contained silver nanowire which is toxic to many bacteria, so its antibacterial ability was enhanced^18^. In that case; nanowires with 175 and 350 ppm silver were utilized to measure the concentration and relevance. It was found that during inflammation, wound healing in the 350 ppm silver nanowire group was better than in the 175 ppm silver nanowire group. However, wound healing in the groups with multiple compound contents (groups E and F) was just the opposite. Therefore, it is suggested that in the early wound inflammation stage, exclusively using 350 ppm silver nanowire will result in a better antibacterial ability.

Also, in this study, it was found that in the early stage of wound healing, PH swelling after absorbing too much tissue fluid played an important role^19, 20^. It not only removed excessive moisture, but also provided physical support to the wound and support for the movement of epidermal cells. However, similarly, when wound healing achieves a certain level, the frequency of using PHs must decrease as well, or excess PHs will oppress the wound. Concerning the process of changing dressings, since adhesion did not occur, in the early stage of dressing application, PHs can be regarded as a good choice.

## Conclusion

In this study use particle hydrogels for the deeper wound can achieve the purpose of filling, as well as providing the effect of wound support while providing the surround cells as a cell migration attachment. In wound dressing change the particle hydrogels easily remove can prevent the wound damage.

## Materials and Methods

### Cell culture

The L929 cell line derived from mouse skin fibroblast cells was maintained in RPMI 1640 medium supplemented with 10% fetal calf serum, 2 mM glutamine, and antibiotics (100 units/mL penicillin and 100 μg/mL streptomycin) at 37 °C in a humidified atmosphere of 5% CO_2_.

### 3-(4, 5-Dimethylthiazol-2-yl)-2, 5-diphenyl tetrazolium bromide (MTT) assay

The effects of different types of hydrogel extracts on cell viability were assessed by an MTT assay. Cells were seeded in 96-well plates at an initial density of 10^5^ cells/mL for 20 hr at 37 °C. After treating cells with hydrogel extracts, 10 μL of MTT (5 mg/mL) was added and incubated for 3 h at 37 °C. The medium was aspirated, and 100 μL of DMSO was added to dissolve the formazan crystals that had formed. Then, the optical density at 570 nm (OD570) was measured by a 96-well plate reader (Epoch Microplate Spectrophotometer, BioTek Inc,). All experiments were performed in triplicate. Cell viability was calculated as follows:

1 $${\rm{The}}\,{\rm{ratio}}\,{\rm{of}}\,{\rm{cell}}\,\text{viability}\,( \% )=\frac{\,({\rm{A}}-{\rm{B}})\,}{({\rm{C}}-{\rm{B}})}\times 100 \% ;$$

where A is the average OD570 of cells treated with different types of hydrogel extracts, B is the average OD570 of control wells (culture medium without cells), and C is the average OD570 of cells no hydrogel extract treatment.

### Lactate dehydrogenase (LDH) assay

Cell death was assayed by quantifying plasma membrane damage. The effects of different hydrogel extracts on cell viability were assessed by an LDH assay. Cells were seeded in 96-well plates at an initial density of 10^5^ cells/mL for 20 hr at 37 °C. After treating cells with the indicated hydrogel extracts, 100 μL/well of a cell suspension was transferred to a sterile 96-well tissue culture plate. To each well was added 100 μl of a solution (25 μL lyophilisate and 75 μL iodotetrazolium chloride-sodium lactate) and incubated for up to 30 min at room temperature while being protected from light

2 $${\rm{The}}\,{\rm{ratio}}\,{\rm{of}}\,{\rm{cell}}\,\text{toxicity}\,( \% )=\frac{({\rm{A}}-{\rm{B}})}{({\rm{C}}-{\rm{B}})}\times 100 \% ,$$

where A is the average OD450 of cells treated with different hydrogel extracts, B is the average OD450 of control wells (culture medium without cells), and C is the average OD450 of cells with Trition X-100 treatment (High control).

### Preparation of PMMA/PVA copolymer agent materials

In this study, the hydrogel particles were synthesized under UV radiation. We used Ciba Irgacure 184 as a photoinitiator, polyglycol 400 monostearate and glycerol as excipient as agents, poly (ethylene glycol) diacrylate as a cross-linking agent.The first step of particle hydrogel precursor synthesis was to mix PVA high polymer solution and cross-linking agent. Second step, the PMMA monomer solution and the photoinitiator were added and mixed evenly as well. The particle hydrogel precursor was use the UV curing machine at 365 nm, irradiation for 10 seconds and the UV light power was 400 W. The particle hydrogels precursor after the UV irradiation the average diameter was 4–5mm/10 μL.

### Original type and Complex particle hydrogels

In this study we used six particle hydrogels including the original (group A), original type mixed with 5% collagen (group B), original type mixed with 350 ppm of silver nanowire (group C), original type mixed with 175 ppm of silver nanowire (group D), original type mixed with 5% collagen, 5% chitosan, and 350 ppm of silver nanowire (group E), and original type mixed with 5% collagen, 5% chitosan, and 175 ppm of silver nanowire (group F). The silver nanowires average length and diameter were 20 μm and 80 nm.

### Use of experimental animal statement

Agricultural Technology Research Institute and Taipei Medical University approving the experiments including any relevant details and confirming all experiments were performed in accordance with relevant guidelines and regulations. The Institutional Animal Care and Use Committee approval were No: 103041 and No: LAC-2013-0060.

### Animal skin sensitization and irritation test model

We used New Zealand white male rabbits of approximately 3 months old and with a body mass of 3.0~4.0 kg (n = 4), in the experiments to evaluate of irritation effects. The detect methods and the erythema and oedema value depend on the ISO 10993–10 tests for irritation and skin sensitization. The particle hydrogels were put on the New Zealand white male rabbits’ back about fill up about the area 2.5 cm * 2.5 cm.

### Animal skin wound model

We use adult male Wistar rats (n = 6, weighing 200–250 g) and male Landrace pigs (n = 6, weighing 30–40 Kg) as our skin wound models. For every rat model there were two wounds, while for pigs, there are six wounds with a 2 × 2-cm area covered with or without the original type hydrogel particles for 7 and 14 days for rats and for 10 and 21 days for pigs. During wound healing, we took photos to compare wound area size between the original hydrogel particle group and the intrasite group. In the hydrogel particle group, the particles were removed with saline solution and easily to measure the wound size. On the contrary, in intrasite group the wound’s secretions with intrasite would form a scab, the wound area size could be measured easily. Perfusion and tissue preparations at 7 and 14 days were for the advanced histochemical/Immunohistochemistry (IHC) analysis. In addition, quantitative histochemical/IHC studies were used to estimate the wound-healing progress. First, animals were deeply anesthetized with 7% chloral hydrate (0.4 mL/kg) and then perfused with 0.9% saline followed by 300 mL of 4% paraformaldehyde in 0.1 M phosphate buffer (PB), pH 7.4. After perfusion, the wound was removed and kept in the same fixative for 2 h. The tissue was then immersed in graded concentrations of sucrose buffer for cryoprotection at 4 °C. Serial 20-µm-thick sections of the wound were cut transversely with (CM3050S, Leica Microsystems, Wetzlar, Germany) the following day.

### Histochemical/IHC stain

The histologic block was fixed in 10% formaldehyde and embedded in paraffin. Tissues in each group were examined microscopically using hematoxylin and eosin (H&E) stain for general histologic observations. Masson’s trichrome stain was applied to observe collagen deposition.

### Statistical analysis

The statistical software, SPSS, was used to calculate the significance according to Student’s t-test. Significance (p value) was accepted as <0.05.
